# Administration time of misoprostol affects fertility rate in artificially inseminated Kivircik ewes with frozen-thawed ram semen

**DOI:** 10.21451/1984-3143-AR2018-005

**Published:** 2018-08-16

**Authors:** Hakan Ustuner, Burcu Ustuner, M. Berk Toker, Selim Alcay, Kamber Demir, Hakan Sagirkaya, Zekariya Nur

**Affiliations:** 1 Department of Animal Science, Faculty of Veterinary Medicine, Uludag University, Gorukle/Bursa, 16059, Turkey.; 2 Department of Reproduction and Artificial Insemination, Faculty of Veterinary Medicine, Uludag University, Gorukle/Bursa, 16059, Turkey.; 3 Department of Reproduction and Artificial Insemination, Faculty of Veterinary Medicine, Istanbul University, Avcılar/Istanbul, 16059, Turkey.

**Keywords:** artificial insemination, Kivircik ewe, misoprostol

## Abstract

The aim of this study was to determine the effects of the administration time of misoprostol (11 h (Miso11) and 6 h (Miso6) before artificial insemination) on fertility rates in Kivircik ewes (control: n = 41, Miso11: n = 32 and Miso6: n = 33) during breeding season. Artificial insemination (AI) was performed 48 h after sponge removal using frozen-thawed semen (150 million sperm per dose in 0.25 ml straws). Estrus synchronization parameters (onset and duration) and lambing rate were evaluated. No significant difference was observed among groups for the estrus onset and duration hours (P > 0.05). The lambing rates in the control, Miso11 and Miso6 groups were 39.0, 62.5 and 54.5%, respectively. There were significant differences among the control, Miso11 and Miso6 groups according to lambing rates (P < 0.05). In conclusion, misoprostol treatment significantly improved fertility in ewes when using frozen-thawed semen in AI. Administration of misoprostol 11 h before AI resulted in a higher lambing rate than that at 6 h before AI; therefore, treatment of misoprostol 11 h before AI can effectively be used.

## Introduction

The genetic progress in farm animals may be possible with the widespread use of artificial insemination (AI) with the highest quality frozen-thawed semen from genetically superior males. Cryopreservation has been reported to cause changes in sperm morphology, including damage to mitochondria, acrosome and sperm tail ( Wooley and Richardson, 1978 ). Therefore, fertility results of deep cervical AI with frozen-thawed semen are low, and obtaining good fertility with frozen-thawed semen requires insemination directly into the uterus ( O'Connell *et al*., 2002 ).

The ewe cervix is a long and fibrous tubular organ. Due to the presence of 4-7 cervical rings in the lumen, its caudal opening provides a physical barrier to external contaminants. The convolute and tortuous structure catheter entrance is more difficult than in the cow ( Wulster-Radcliffe *et al*., 2004 ; Kershaw *et al*., 2005 ; Leethongdee, 2010 ; Aral *et al*., 2011 ).

The rate of AI achievement in sheep may vary between 76 and 10% ( Windsor, 1995 ; Kershaw *et al*., 2005 ) depending on breeds. Such different success of AI among individual ewes may be explained by the great variation in cervical anatomy among animals ( Kershaw *et al*., 2005 ). There are considerable differences between species, even each breed of sheep regarding the complexity of cervical rings, organization of the inner and outer orifices, length and complexity of the cervical lumen and anatomical relationships with the uterine body and vagina ( Leethongdee *et al*., 2007 ).

We know that the cervix functions through the remodeling of the extracellular matrix components, such as dissociation of collagen fibers, degradation of proteoglycans and the release of glycosaminoglycans (GAGs), specifically during late gestation, parturition and estrus ( Leppert, 1992 ; Leethongdee, *et al*., 2010 ). Hyaluronan is the predominant GAG, whose synthesis is stimulated by Prostoglandin E2 in the sheep cervix ( Dobson, 1988 ) and its concentrations vary during the estrus cycle ( Kershaw-Young *et al*., 2009 ). Hyaluronan content of the cervix has been reported to be the highest prior to the preovulatory LH surge of the estrus cycle, when there is also a degree of natural relaxation of the cervix ( Leethongdee *et al*., 2007 ). Recent researches showed that intravaginal application of a PGE1 analogue, such as misoprostol, after estrus synchronization can induce cervical ripening, which has a direct correlation with the rate of resulted pregnancy, and is greater when the catheter reaches the uterus ( Leethongdee *et al*., 2007 ; Horta *et al*., 2010 ).

The misoprostol is generally administered 6 h before artificial insemination to increase the cervical depth penetration in different sheep breeds ( Aral *et al*., 2011 ). A longer time lag between treatment and AI may be required to avoid drugs interference on semen performance and to allow maximal biochemical and structural transformations of the cervix to occur before AI. However, limited reference information exists regarding the effect of misoprostol on the fertility rates of Kivircik ewes. Thus, the objective of this study was to determine the effects of the administration time of misoprostol (6 and 11 h before artificial insemination) on fertility rates following fixed-time artificial insemination.

## Materials and Methods

The Scientific Ethical Committee (Uludag University, Bursa, Turkey, No: 2012-14/3) approved all protocols related to the experimental setup and evaluation techniques. The experiment was carried out at an experimental commercial farm, (38°28′52″N latitude and 28°8′21″E longitude) in Manisa, Turkey, during the breeding season (July) under natural lighting. In this study, 106 clinically healthy Kivircik ewes (2 to 4 years old), 5 fertile Kivircik rams for semen collection and 6 teaser rams for estrus detection were used. The teaser rams were used rotationally (changed daily). The ewes were group- housed in an open barn. All ewes were fed with a commercial concentrate diet with hay and water provided ad libitum. Ewes were kept away from the rams to prevent voluntary mating. In the meantime, a teaser ram was introduced to the flock for a short time (1 h) on each occasion (once or twice weekly) to determine the presence of estrus cyclicality in season. Body weights and condition scores of these animals were recorded prior to the experiment. Ewes weighing between 45 to 55 kg with good body conditions (BCS:3 to 3.5) were used.

### Intra-cervical application of misoprostol

All ewes were treated with intravaginal sponges containing 40 mg of progesterone (40 mg of FGA® Fluorogestone Acetate, Intervet Productions SA, Lyons, France) for 12 days. The injection of 250 µg/ml of PGF2α analog cloprostenol (1 ml of Juramate, Jurox Pty Ltd, Australia) was applied 24 h before sponge removal. An i.m injection of 400 IU of PMSG (Intervet Productions SA., Lyons, France) was given at sponge removal to induce and synchronize heat. Ewes (n = 106) were divided into three groups. Ewes in the control group (n = 41) were not treated, but the procedure was carried out without administering a tablet. Ewes in groups 11 h (Miso11, n = 32) and 6 h (Miso6, n = 33) had one pill of Cytotec (200 µg of misoprostol, Cytotec^®^; Pfizer, England) inserted into the vagina before AI, corresponding to 37 and 42 h after sponge removal, respectively. For the misoprostol treatment; ewes were restrained in a yoke fitted with side bars to minimize lateral and forward movements, with their hindquarters raised about 4 inches. The perineum was cleaned with a disinfectant wipe and, using a vaginal speculum, misoprostol tablet was placed at the external os of the cervix with the help of long forceps. Each ewe was restrained during the administration of misoprostol tablet for no more than 5 min to minimize the stress on the ewes.

### Observation of estrus signs

Ewes were monitored every 6 h for 1 h, starting from 12 to 80 h after sponge removal, for both the signs of estrus behavior and their durations with the aid of teaser rams. Ewes were considered in estrus when they allowed the male to mount. Estrus duration was defined as the time elapsed between the first and last accepted mount within the same estrus period.

### Semen collection, freezing and artificial insemination

Five rams with previously proven fertility were used for semen collection with the electroejaculator (Minitube-Germany). To collect semen, each ram was physically restrained, and a lubricated probe was inserted into the rectum with downward pressure being maintained on the front of the probe, so the electrodes remained near the upper portion of the ampullary region ( Ustuner *et al*., 2016 ). When the electrostimulation was stopped briefly, further massage was applied with the probe. This cycle was repeated until 1.5-2 ml of semen was collected (usually 3-4 electrostimulations). Collected semen was placed in a warm water bath (30°C) and evaluated immediately for consistency, wave motion (0-5 scale) and percentage of motile spermatozoa (%). Ejaculates with a thick consistency, only 0.5-1.5 ml of sperm with rapid wave motion (2-5 on a 0-5 scale), and >70% initial motility were pooled and diluted with a Tris-based extender (20% egg yolk; v/v) to a final concentration of 1:5 (semen:extender) in 6% glycerol using a two-step dilution method ( Aisen *et al*., 2000 ). The semen samples were frozen in 0.25 ml straws in liquid nitrogen vapor using a Nicool Plus PC freezing machine (Air Liquide, Marne-la-Valle´e Cedex 3, France), and then they were plunged into liquid nitrogen at -196°C.

The straws were thawed at 37°C for 30 sec in a water bath for insemination of ewes. In total, 0.25 ml corresponding to 150 million sperm cells with at least 45 to 50% progressive motility was delivered to each ewe. Intra-cervical artificial insemination was performed 48 h after sponge removal, with 0.25 ml straws. Cervical AI was considered when semen was deposited at least after the first cervical ring.

### Lambing rate

Lambing rates (percentage of ewes lambing) were recorded following 150 ± 5 days of inseminations. Lambing rates were calculated as follows: lambing rate = (lambs born/ewes inseminated) × 100 ( Zeleke *et al*., 2005 ).

### Statistical analysis

The onset of estrus and duration were subjected to an analyses of variance (one-way ANOVA), and the differences among means were tested for significance by Tukey’s test. Lambing rates were analyzed using the chi-square test.

## Results

The results in terms of estrus response for the time to the onset and duration of the induced estrus and lambing rates are set out in Table 1 . No significant difference was observed among groups for the estrus onset and duration hours (P > 0.05).

**Table 1:** Mean (±SE) onset and duration of oestrus (h) and lambing rate (%) of ewes in relation to administration of misoprostol 11 and 6 h prior to insemination (control *vs* . treatment).

Group	n	Onset	Duration	Lambing rates (%)
Control	41	38.25 ± 9.85	19.13 ± 6.72	16 (39.02)^c^
Miso11	32	44.89 ± 10.83	17.33 ± 9.01	20 (62.50)^a^
Miso6	33	41.22 ± 8.73	20.87 ± 9,72	18 (54.55)^b^

Data were presented as mean ±SE. ^a,b,c^Different superscripts in the same column indicate significant differences among groups (P < 0.05).

**Figure 1:**
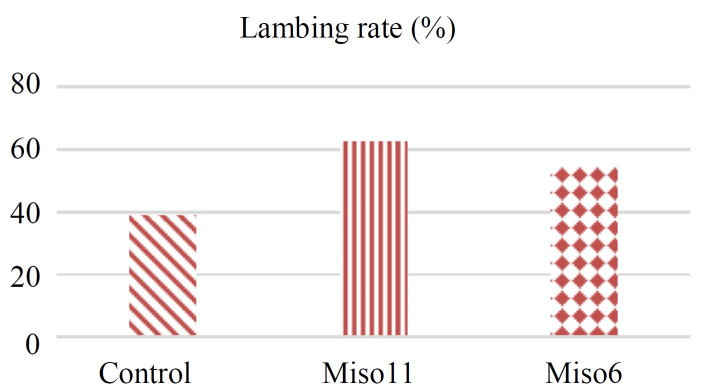
Lambing rate (%) of ewes in relation to administration of misoprostol 11 and 6 h prior to insemination (control *vs*. treatment).

As shown in Table 1 and Fig. 1 , the lambing rate was 39.0% in control group, 62.5% in the Miso11 group and 54.5% in the Miso6 group. There were significant differences among the control, Miso11 and Miso6 groups according to lambing rates (P < 0.05). The 7.9% improvement in Miso11 fertility when compared to the Miso6 fertility depended on a more comfortable passage of the cervix.

Table 1 . Onset and duration of estrus (h) and lambing rate (%) of ewes in relation to the administration of misoprostol 11 and 6 h prior to insemination (control *vs*. treatment).

Figure1 . Lambing rate (%) of ewes in relation to administration of misoprostol 11 and 6 h prior to insemination (control *vs*. treatment).

## Discussion

Artificial insemination in sheep has two major limiting factors: the poor quality of frozen-thawed ram semen and the convoluted anatomy of the sheep cervix that does not allow transcervical passage of an insemination catheter ( Falchi *et al*., 2012 ). Therefore, this investigation was conducted to determine the effect of vaginal administration time of prostaglandin E1 analogue, misoprostol, for trans-cervical artificial insemination by using frozen-thawed semen in Kivircik ewes during breeding season.

Responses to intravaginal sponges have varied according to breed, protocol, co-treatment, management, mating system and geographical location which is known to influence this period ( Evans and Maxwell, 1987 ; Gordon, 1997 ). The mean estrus parameters did not differ (P > 0.05) among the groups. In this study the time to estrus onset following the withdrawal of a sponge were 38, 44 and 41 h in the control, Miso11 and Miso6 groups, respectively, which were longer than the 28 h found by Rekik *et al*. (2016) and similar to that found by Zeleke *et al*. (2005) and Zonturlu *et al*. (2011) . Estrus duration time was shorter than reported by Ustuner *et al*. (2007) and similar to those reported by Zeleke *et al*. (2005) and Zonturlu *et al*. (2011) .

Misoprostol influences the smooth muscle activity of the genital tract, including cervical muscle activity, which is important for sperm progression ( Hawk, 1983 ; Horta *et al*., 2010 ). Prostaglandins of the E series have been shown to induce collagen breakdown and softening of the cervical tissue structure, a mechanism known to be associated with local production of PGE and glycosaminoglycans ( Ellwood *et al*., 1980 ; Ledger *et al*., 1983 ). It was recently proposed that prostaglandin E2 selectively binds to EP2 and EP4 prostaglandin E2 receptors, stimulating hyaluronan (HA) synthesis, which may cause remodeling of the cervical extracellular matrix and culminating in cervical relaxation ( Kershaw-Young *et al*., 2009 ). In this study, the significantly higher lambing rate after the administration of misoprostol when compared to the control group strengthens the importance of the depth of insemination when using frozen-thawed semen and this was in accordance with Barbas *et al*. (2013) . Barbas *et al*. (2013) reported that lambing rates of Saloia ewes treated with misoprostol after 48 h of synchrony (6 h before AI) were 41.4 and 30.2% in treatment and control groups, respectively. The lower lambing rate of the control group than the Miso6 and Miso11 groups could be explained according to Leethongdee *et al*. (2007) , who reported that penetration of the cervix was least at the time of sponge removal. In addition, a maximum relaxation of the cervix occurs 72 h after sponge removal, which is too late for the correct fixed-time artificial insemination.

The optimum time for artificial insemination of ewes is not at 72 h after sponge removal ( Leethongdee *et al*., 2007 ). Highest fertility is achieved when ewes were inseminated 48 -54 h after sponge removal ( Evans and Maxwell, 1987 , Ustuner *et al*., 2007 ). Therefore, the recommended treatment time of misoprostol for cervical relaxation in sheep is 6 h before artificial insemination ( Leethongdee *et al*. 2007 , Horta *et al*., 2010 , Aral *et al*., 2011 ).

Frozen-thawed semen is a critical determinant of fertility, due to the significantly reduced lifespan of frozen-thawed semen in the female reproductive tract ( Kumar and Naqvi, 2014 ). Considering the life span of the frozen sperm, it was planned that the application of misoprostol would be done 11 h before fixed-time artificial insemination to cervical relaxation.

This was the first time that the effect of misoprostol treatment 11 h before fixed-time artificial insemination with frozen-thawed semen of Kivircik ewes upon fertility was studied. In our experiment, ewes were administered misoprostol 11 h before artificial insemination, which resulted in a significantly higher percentage of lambing rates than the Miso6 or control groups (P < 0.05). Although the difference of 7.9 percentage units between Miso11 and Miso6 is small in magnitude, it is certainly of practical and economic significance.

In a previous work ( Rashidi and Cedden, 2013 ), the administration of misoprostol to ewes in estrus, 48 h after synchronization (3-4 h before insemination) resulted in a 44.87% lambing rate, which is lower than the values obtained in the present study (Miso11 = 62.5% and Miso6 = 54.5%). Differences in fertility rates after misoprostol application in sheep may originate from the artificial insemination technique and quality of frozen-thawed sperm.

This study demonstrated that misoprostol treatment significantly improves fertility in ewes when using frozen-thawed semen in AI. Administration of misoprostol 11 h before AI resulted in a higher lambing rate than at 6 h before AI. Therefore, treatment of misoprostol 11 h before AI can effectively be used.
